# Inclusive Fees for Private Patients

**Published:** 1919-04-26

**Authors:** 


### Inclusive Fees for Private Patients.

### St. Chad's Hospital, Birmingham.

In view of a paragraph which appeared recently in The Hospital (March 22, p. 538) regarding the above institution, the following further facts have reached us which define in some detail the precise objects of the hospital. It is believed that the institution is the only one of its nature in the United Kingdom. So that there could be no resort to charity on the part of the patients, the hospital has been built by a limited liability company formed by a group of Birmingham consultants with the assistance of several business men, to supply special medical and surgical treatment, under proper conditions, for that section of the community which, not of the voluntary hospital class, is yet unable to afford the expense which such treatment ordinarily involves.

Patients are recommended for admission and fees fixed by the members of the medical staff only, whose qualifying status must be that of a consultant holding a position on the staff of a Birmingham hospital. The fees are arranged on an inclusive basis—*i.e.* one charge to cover nursing, medical, and operative treatment. Every branch of medicine and surgery is represented, and a feature of the hospital is that any member of the medical staff can obtain the opinion and assistance of any of his colleagues without further charge to the patient. Constant observation is possible by the help of a resident medical staff, and a matron is directly responsible to the Medical Committee for the nursing. During the existence of the hospital (which covers a period of four years) about 70 per cent. of the patients admitted have paid inclusive fees varying from five to seven guineas per week. A number of these patients during their stay obtained a consultation between a physician and a surgeon, received an *x*-ray examination, a pathological investigation, and subsequent operation, which treatment was covered by the inclusive charges above mentioned. Dr. Hemingway, a woman doctor, is the resident medical officer. The medical staff consists of twenty-one members.

According to the 1918 report of the Edgbaston Private Nursing Home, Ltd., a final dividend of 6 per cent. was paid on the preference shares, and a dividend of 10 per cent. on the ordinary shares. The nominal capital of the company is £12,000.

